# Bacterial contamination of automated MRI contrast injectors in clinical routine

**DOI:** 10.3205/dgkh000321

**Published:** 2019-05-17

**Authors:** Juliane Goebel, Joerg Steinmann, Evelyn Heintschel von Heinegg, Tobias Hestermann, Kai Nassenstein

**Affiliations:** 1 Department of Diagnostic and Interventional Radiology and Neuroradiology, University Hospital Essen, Germany; 2 Institute of Clinical Hygiene, Medical Microbiology and Infectiology, Paracelsus Medical University, Hospital Nuernberg, Germany; 3 Institute of Medical Microbiology, University Hospital Essen, Germany

**Keywords:** MR imaging, automated contrast injector, contrast agent, hygiene, bacterial contamination

## Abstract

**Aim:** To quantify the frequency of bacterial contamination of the injected contrast agent/saline solution by an automated contrast injection system, and to evaluate whether usage of a novel tube system can reduce it.

**Methods: **For bacterial contamination quantification two identical automated piston pump MRI contrast injectors were used in combination with a standard tube system. 3–5 ml of the contrast agent/saline solution was collected from the system prior to its connection to the patients’ venous cannula in 104 consecutive patients. To test, whether a novel tube system reduces contamination, a tube system with shielded screw connections was used with the same contrast injectors and contrast agent/saline samples were collected in further 101 patients. Specimens were microbiologically analyzed. Frequencies of contamination were compared using Fisher exact test.

**Results:** With the standard tube system, bacterial contamination was observed in 5.8% (6 out of 104 specimens). With the novel tube system, contamination was observed in 2.0% (2 out of 101 specimens, p=0.280). *Staphylococcus epidermidis* was the most common germ (5 cases) followed by *Micrococcus luteus* (2 cases) and *Oligella ureolytica* (1 case).

**Conclusion: **Bacterial contaminations of MRI contrast injectors occurred in a non-negligible frequency especially with *S. epidermidis*. A trend towards reduced bacterial contamination was seen when a novel tube system with shielded screw connections was used.

## Zusammenfassung

**Ziel:** Es sollte die Häufigkeit der bakteriellen Verunreinigung des injizierten Kontrastmittel-/Kochsalzgemisches in der klinischen MRT-Routine erfasst und untersucht werden, ob selbige durch Verwendung eines optimierten Kontrastmittelinjektorsystems reduziert werden kann.

**Methoden: **Es wurde ein automatisches Kolbenpumpen-Kontrastmittelinjektorsystem kombiniert mit einem Standard MRT-Infusionsschlauchsystem verwendet und vor der Ankopplung an die venöse Verweilkanüle von 104 konsekutiven Patienten 3–5 ml des Kontrastmittel-/Kochsalzgemisches entnommen. Zudem wurde bei weiteren 101 Patienten ein optimiertes Infusionsschlauchsystem mit versenkten Schraubenkontakten verwendet und ebenfalls 3–5 ml des Kontrastmittel-/Kochsalzgemisches gesammelt. Die Proben wurden mikrobiologisch analysiert und die Kontaminationshäufigkeit mittels Fisher’s Exact Test verglichen.

**Ergebnisse: **Beim Standard-Infusionsschlauchsystem fand sich in 5,8% (6 von 104 Proben) und beim optimierten Infusionsschlauchsystem in 2,0% (2 von 101 Proben) eine bakterielle Verunreinigung (p=0.280). Am häufigsten war *Staphylococcus epidermidis* anzüchtbar (n=5), seltener *Micrococcus luteus* (n=2) und *Oligella ureolytica* (n=1).

**Schlussfolgerungen: **Eine bakterielle Kontamination des Kontrastmittelinjektorsystems, speziell mit *Staphylococcus epidermidis* trat in nicht unerheblicher Frequenz auf und konnte durch die Verwendung eines optimierten Infusionsschlauchsystems tendenziell gesenkt werden.

## Introduction

Although magnetic resonance imaging (MRI) provides an excellent soft tissue contrast, intravenous contrast media application is necessary in many cases (e.g. for angiography, analysis of vascularization or perfusion) to improve the diagnostic power of MRI [1]. In order to achieve a well-defined contrast injection with regard to contrast media amount, flow, as well as timing the use of automated injector systems is, similar to computed tomography, standard in clinical MRI routine. Even though different systems are available from diverse vendors, all automated injection systems have in principle a similar buildup: Contrast media as well as saline solution are stored in a container and are pumped by a piston pump or roller pump via a tube system into the patients’ indwelling venous cannula. Typically the tube system, which connects the pump to the venous cannula, is constructed from two tubes, which are connected via a check valve, so that only the distal tube – the patient line – as well as the check valve has to be replaced between consecutive patients. However, since the system is used in multiple patients it entails the risk of bacterial contamination of the injected contrast agent/saline solution [2], [3], [4], [5], and, therefore, a potential risk of nosocomial infections exists [6], [7], [8], [9]. Thus, the aims of the present study were to quantify the frequency of a bacterial contamination of the contrast agent/saline solution in a pistol pump automated injection system in clinical routine, and to evaluate whether a novel tube system with shielded screw connections can reduce the potential risk of bacterial contamination.

## Methods

The study was approved by the local ethic committee.

### Contrast media and contrast injector

Automated contrast injections were performed using a Medrad^®^ Spectris Solaris^®^ EP MR injection system (Bayer Vital GmbH, Bayer HealthCare, Leverkusen, Germany), and Gadoterate meglumine (Dotarem, Guerbet GmbH, Sulzbach, Germany) was used as contrast media in all cases. All patients received a standard dose of 0.1 mmol Gadoterate meglumine per kg body weight at a flow rate of 2 ml/sec followed by 20 ml physiological saline solution at the same flow rate.

### Standard contrast injector and tube setting

**Figure 1:**
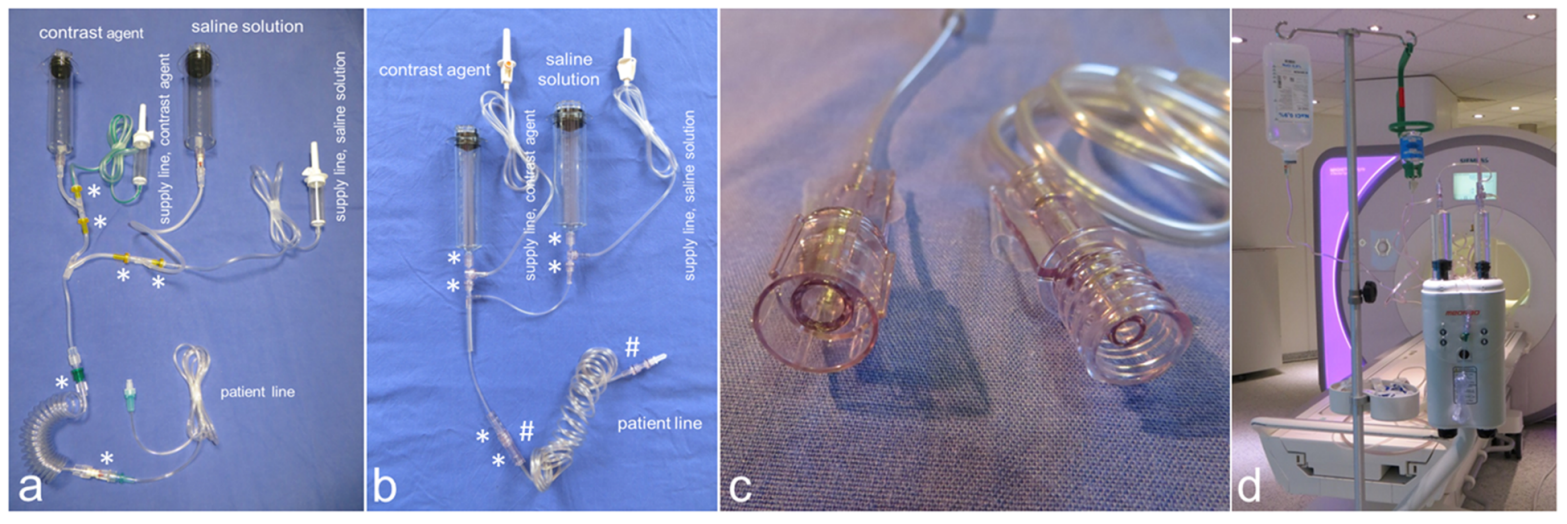
MRI tube systems Standard tube system (a) composed of a syringe for contrast media, a syringe for saline solution, a T-shaped connector tube with re-filling lines, a spiral infusion line, and the patient line. Novel tube system (b, d) composed of a syringe for contrast media, a syringe for saline solution (similar to a), a fluid transfer set for re-filling, a T-shaped connector tube, and a spiral patient line with shielded screw connectors (c; b #). Check walves within the tube system are marked by an asterisk (*).

As standard injection system setting only the syringes of the disposable MRI kit for 65/115 MR injector system (catalogue number: SSQK65/115VS; Bayer Medical Care B.V., Maastricht, The Netherlands) were used – one syringe for contrast media with a capacity of 65 ml and one syringe for saline with a capacity of 115 ml – and were connected by a T-shaped connector tubing with integrated check walves and separate supply lines for contrast agent and saline solution (article number: 314100-100; Medton AG, Saarbruecken, Germany, approved for multiple use up to 8 hours) to a spiral infusion line (Figure 1a (Fig. 1)). This system was connected by a check valve (R-Lock, Codan Pvb Medical GmbH, Lensahn, Germany) to an infusion line (Original Perfusor Line, Braun Melsungen AG, Melsungen, Germany), which again had been connected to the patients’ indwelling venous cannula. While the check valve and the infusion line were replaced for each patient as advised by the producer, the syringes, the T-shaped connector tube and the spiral infusion line were used in a multi-session setting up to 8 hours. The supply line for contrast agent was connected to a 100 ml glass multi-dose vial containing 0.5 mmol/ml Gadoterate meglumine (Dotarem, Guerbet GmbH, Sulzbach, Germany); the supply line for contrast agent was connected to a 500 ml vial containing physiological saline solution (B. Braun Melsungen AG, Melsungen, Germany). The technicians were instructed to perform alcohol-based hygienic hand disinfection (Descoderm, Dr. Schumacher GmbH, Melsungen, Germany) prior to all manipulations on the injection system or the tubing.

### Novel contrast injector and tube setting

With regard to a potentially hygiene optimized setting of the injector again only the syringes of the disposable MRI kit for 65/115 MR injector system (catalogue number: SSQK65/115VS; Bayer Medical Care B.V., Maastricht, The Netherlands) were used. For tubing the tube system of a Medrad^®^ Stellant multi-patient kit (catalogue number: SDS MP1; Bayer Medical Care B.V., Maastricht, The Netherlands) was used, which is approved for multiple use up to 12 hours and which features shielded screw connections minimizing the risk of bacterial contamination of the connectors (Figure 1b,c (Fig. 1)). Beside integrated check walves, this tube system contains a fluid transfer set, placed between the syringes and the T-shaped connector tubing, which allows re-filling of the syringes from a connected storage container. Similar to the standard tube setting the T-shaped connector tubing was connected to a spiral single patient disposable infusion line (catalogue number: SPD250; Bayer Medical Care B.V., Maastricht, The Netherlands), which features shielded screw connections to the T-shaped connector tubing and two check valves in series. Similar to the standard procedure the supply lines were connected to the vials with contrast media and physiological saline solution and only the patient line was replaced between consecutive patients, while the rest of the system was used during the 8 hours working day without replacement. As always, the technicians were instructed to perform hand disinfection prior to any manipulation at the injection system or at the tubing.

### Specimen sampling

Specimen sampling was consecutively performed on two MR systems (Magnetom, Siemens Healthcare, Erlangen, Germany), using the above mentioned automated injection systems daily alternating, until at least 100 specimens were collected in both study arms. During the 8 hours working day the technicians collected 3–5 ml of the contrast agent/saline solution from the injection system after they connected the new patient line and before connecting this tube to the patients’ venous cannula. The specimens were collected in sterile laboratory vials (Roehre 13 mL, Nuembrecht, Germany), and were temporarily stored in a laboratory refrigerator at 4°C up to 12 hours before transmission to the microbiology laboratory. The vials were labeled with the day of sampling and a consecutive number. After 21 days the target number of collected specimens was achieved in both study arms.

### Patients and their MRI examinations

Using the standard contrast injector and tube setting, 104 unselected patients (52 women and 52 men; mean patient age, 53.3 years (range, 6–84 years)) were examined. Using the novel contrast injector and tube setting, 101 unselected patients (54 women and 47 men; mean patient age, 53.0 years (range, 5–93 years)) were examined. There was no relevant difference in age (p=0.886) or sex (p=0.620) between both groups. Moreover, no relevant difference between examined body regions existed between both groups (standard contrast injector group: head, 63; neck, 9; spine, 11, thorax, 1; abdomen, 13; pelvis, 5; upper limb, 1; lower limb, 1; the novel contrast injector group: head, 58; neck, 6; spine, 5; thorax, 4; abdomen, 13; pelvis, 9; upper limb, 1; lower limb, 5).

### Microbiological analysis

For microbiological analysis 1 ml of the contrast medium/ saline specimen was inoculated into BacT/Alert bottles iAST and iNST (bioMérieux, Nuertingen, Germany) for automated microbial detection based on the colorimetric detection of CO_2_ produced by growing microorganisms in Casein Soja-Pepton bouillon. The aerobic bottle was incubated at 22±1°C for seven days and the anaerobic bottle at 32±1°C also for seven days based on the method described in the European Pharmacopoeia for sterility testing [10]. Automatic readings were performed every 10 minutes. In case of positivity, aliquots of the bouillon were plated for culturing on solid media (Columbia blood agar, Chocolate agar, MacConkey agar, Universal BEER agar, Schaedler anaerobe agar, Sabouraud agar, all from Oxoid, Wesel, Germany). The time from start of incubation of the bottles to the signal of the system of positivity (time to positivity) was calculated. Identification of microorganisms was performed with the mass spectrometry VITEK MS, or the semi-automated platforms VITEK 2 (bioMérieux, Nuertingen, Germany) and WalkAway (Beckman Coulter, Krefeld, Germany).

### Statistical analysis

For statistical analysis SPSS Statistics 22 (IBM Corporation, Armonk, NY, USA) was used. The frequency of microbial contamination was calculated for each tube system used. A Fisher's exact test was used for statistical analyses of the observed frequencies. A p value lower than 0.05 was considered statistically significant.

## Results

**Figure 2:**
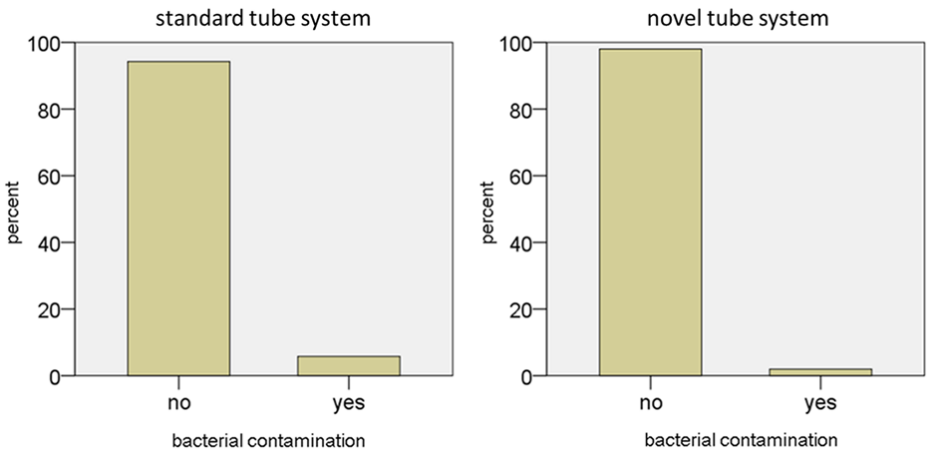
Frequency of bacterial contamination of both MR tube systems

Using the standard contrast injector and tube setting 6 of 104 (5.8%) collected contrast agent/saline solution specimens were microbiologically contaminated (Figure 2 (Fig. 2)). 

In 4 cases *Staphylococcus epidermidis* was identified as the contaminating microorganism, while *Micrococcus luteus* and *Oligella ureolytica* were found each in one more case. Detailed analyses showed that the contaminations occurred at every MRI participating in this study, and that no consecutive specimens had been contaminated: Three samples contaminated with *Staphylococcus epidermidis* were collected on the same day but on two different MR scanners. In two cases the following samples were not contaminated, in one case the contaminated specimen was the last sample of the day. The fourth sample contaminated with *Staphylococcus epidermidis* was collected on a different day and the following specimens were not contaminated. The two specimens contaminated with *Micrococcus luteus* and *Oligella ureolytica* were collected on the same day at the same MR site, but not in immediate succession, and in both cases the following samples were not contaminated. The time to positivity was for all samples >24 hours; range: 26–104.6 hours (Table 1 (Tab. 1)), indicating a low germ load.

Using the novel injector tubing only 2 of 101 (2.0%) collected contrast agent/saline solution specimens were contaminated (Figure 2 (Fig. 2)). *Staphylococcus epidermidis* and *Micrococcus luteus* were identified as the contaminating bacteria, each in one case. The two contaminated specimens were collected on different days and both times the following samples showed no bacterial contamination. Statistical analyses revealed no significant difference between both tube systems (p=0.280).

Neither patients examined by use of the standard infusion system nor patients examined by use of the optimized infusion system suffered from any bloodstream infection up to 7 days after the MR examination.

## Discussion

Although significant improvements have been made to reduce healthcare-associated infections (HAI) a recent study from 2014 estimated that in 2011 722,000 HAIs occurred in U.S. acute care hospitals, and that 75,000 patients with HAIs died during hospitalization [11]. Beside direct or indirect contact transmissions, which are by far the most important transmission routes for HAIs, common vehicle transmission is a further relevant transmission route. Despite the fact that MR contrast injector systems can serve as “vehicles” for microorganisms little is known about the frequency of bacterial contamination of these systems in clinical routine. This is astonishing against the background that in the U.S. in 2014 110 MRI examinations where performed per 1,000 inhabitants, which provides insight how relevant this transmission way might be [12].

Our study revealed that automated contrast injection systems used for MRI show a bacterial contamination in up to 5.8% of injections in clinical routine (6 contaminations in a total of 104 specimens). Comparable rates of bacterial contamination were reported by Nakataki et al., who found 5.7% (15 out of 265) of their investigated infusion set needles bacterially contaminated, and by Trautmann et al., who found 7.8% (4 out of 51) of their investigated intravenous infusion fluids from infusion bottles bacterially colonized [7], [8]. Moreover, our study showed that bacterial contamination of the contrast media/saline solution was caused primarily by microbes from the skin flora, first and foremost *Staphylococcus epidermidis*. This perfectly corresponds to the spectrum of bacterial contaminants reported by Nakataki et al. [8] and Trautmann et al. [7], who reported about contamination by bacteria commonly present on hands with a predominance of *S. epidermidis*.

*S. epidermidis*, a regular part of the microbiota of the human skin, belongs to the coagulase-negative staphylococci (CoNS), which had been classified historically as being less or nonpathogenic [13], [14]. CoNS represent one of the major nosocomial pathogens, with *S. epidermidis* being one of the most significant species, and *S. epidermidis* is now seen as an important opportunistic pathogen [12]. Demographic and medical developments creating more elderly, multimorbid, and immunocompromised patients and the increasing use of inserted or implanted foreign bodies have contributed to the progressively increasing importance of CoNS in health care. Since *S. epidermidis* has the ability to adhere to abiotic surfaces like medical implants and to format biofilms on these, the most important clinical entity associated with *S. epidermidis* are foreign body-related infections [12]. Even though *S. epidermidis* infections are predominantly subacute or chronic and only rarely develop into life-threatening diseases, *S. epidermidis* infections should not be dismissed, since their treatment is complicated by specific antibiotic resistance genes, the formation of biofilms that have intrinsic resistance to antibiotics, and molecular determinants facilitating *S. epidermidis* immune evasion. Taking this into account, the observed contaminations of the contrast injector’s tubings with *S. epidermidis* may be considered as clinically relevant.

In two cases *Micrococcus luteus* and *Oligello ureolytica* were detected in the contrast media/saline solution. *M. luteus* is generally regarded as an ubiquitous occurring microbe and as a harmless saprophyte that inhabits or contaminates the skin [15], [16]. Only in cases of severe immunodeficiency *M. luteus* has been associated with various infections including pneumonia, sepsis, endocarditis, intracranial abscesses, meningitis, and foreign body-related infections [16]. The same applies to *O. urealytica*, which is an aerobic gram-negative coccobacillus found as a commensal organism in human urinary tracts [17]. Of the few reported cases of symptomatic infections, all occurred as opportunistic infections in patients with immunosuppression such as malignancy, HIV, or newborn status [18]. Therefore, the observed contaminations with *M. luteus* and *O. ureolytica* seem to have a low risk for nosocomial infection.

Based upon the observed bacterial spectra and the relatively long time-to-positivity, it is obvious that the microbiological contaminations were caused by manipulation at the injector’s tubing system without or with insufficient prior hand disinfection. Looking when and where the contamination occurred, it is apparent, that the contaminations happened intermittently at the screw connections of the tubing system during the replacement of the patient line. Fortunately, our study shows that the contaminations of the patient lines resulted not in a clinically relevant “ascending” microbiological contamination of the rest of the injection system (e.g. syringes) since a microbiological contamination was not detectable in consecutive samples, which can be explained by the use of multiple check walves within both tubing systems. Moreover, from the fact that no consecutive samples had been microbiologically contaminated it can be concluded, that the filling of the syringes caused no bacterial contamination.

Our results suggest that the bacterial contamination of the injector’s tube system can be reduced by using a system with shielded screw connections (Figure 1b,c (Fig. 1)) that reduces the risk of accidental contact to the connecting component. However, our study revealed that shielded screw connections only reduce, but not eliminate the risk of microbiological contamination. Therefore, regular staff training with respect to necessary hygiene standards, especially regular hand disinfection/strict aseptic techniques [5], [9] seems to be key actions to reduce microbiological contamination of MRI contrast injector systems.

Even though we observed no bloodstream infections in our study, this finding does not imply that the observed bacterial contaminations are negligible, since the number of bacterial contaminations observed in the current study had been too small to estimate the risk of a symptomatic infection.

Albeit the present study indicates that the described use of the MR injector system up to 8 hours is safe with respect to the fact that no bacterial contaminations occurred in consecutive patients, it must be stated that the described use implies an off-label use since the syringes are labeled as “do not reuse”. Although this does not mean that the syringes have to be changed after every patient, but a refilling of the syringes is not allowed even when (like in the present study) a T-shaped connector tubing with separate supply lines explicitly manufactured for this purpose is used. However, it must be noted that, in contrast to similar CT injector systems, currently no syringes are available which are approved for multiple usage.

The present study has several limitations. First and foremost, the single center study design limits the generalizability of our results. Secondly, the sample size has been rather small, which may have caused the lacking statistical significance between the results of both injectors’ setups. Thirdly, a contamination during specimen collection or a secondary contamination of the collected specimen during microbiological analyses could not be excluded with certainty even though the microbiological analysis of the collected samples was performed under aseptic conditions in a cleanroom. Fourthly, since the technicians had been aware of the study, they may have paid more attention towards hygienic standards during the sample collection, than they possibly would have done in the case of unobserved clinical routine, what may result in an underestimation of the microbiological contamination. However, we tried to minimize this bias by waiving any additional examinations (e.g. microbiological examinations of the technicians’ hands), which may had resulted in an aberrance from clinical routine. Finally, our study allows no conclusion concerning the question in which percentage a bacterial contamination of the MR injector systems results in a HAI, due to the small sample size of our study.

## Conclusions

In conclusion, the present study shows that bacterial contaminations of the MRI contrast injector’s tube system occur in a non-negligible frequency especially with *S. epidermidis*, which can cause nosocomial infections. Moreover, in our study bacterial contaminations occurred solely at the screw connections of the tubing system during the replacement of the patient line, while the rest of the contrast injector system showed no bacterial contaminations during its 8 hours use. Furthermore, our study indicates by trend that the use of a novel tube system with shielded screw connections can reduce the risk of bacterial contamination.

## Notes

### Competing interests

The authors declare that they have no competing interests.

## Figures and Tables

**Table 1:**
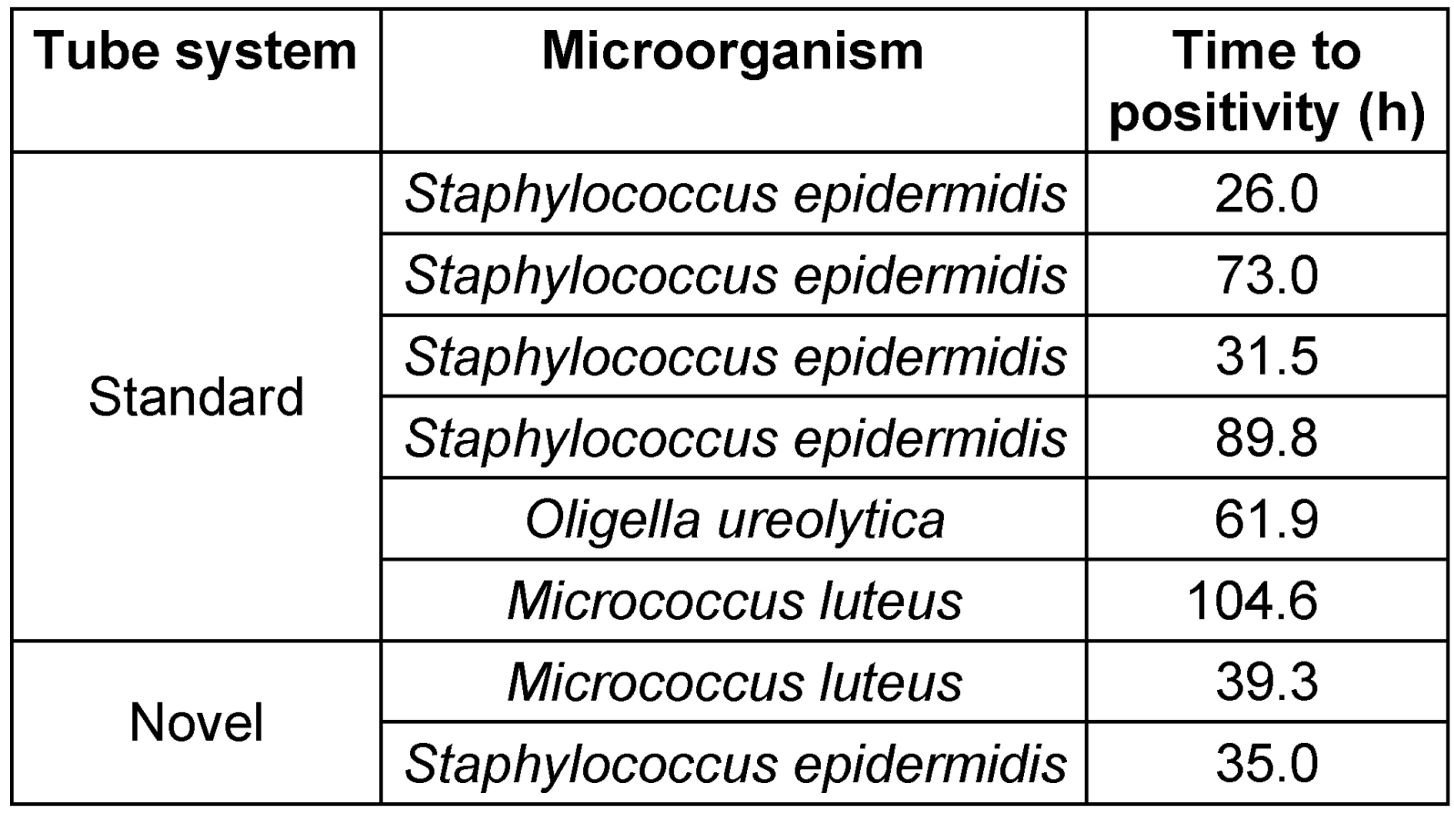
Time to positivity of all samples with microbial growth
